# Enhanced Photoelectrochemical Behavior of H-TiO_2_ Nanorods Hydrogenated by Controlled and Local Rapid Thermal Annealing

**DOI:** 10.1186/s11671-017-2105-x

**Published:** 2017-05-05

**Authors:** Xiaodan Wang, Sonia Estradé, Yuanjing Lin, Feng Yu, Lluis Lopez-Conesa, Hao Zhou, Sanjeev Kumar Gurram, Francesca Peiró, Zhiyong Fan, Hao Shen, Lothar Schaefer, Guenter Braeuer, Andreas Waag

**Affiliations:** 1 Institute for Semiconductor Technology, TU Braunschweig, Hans-Sommer-Strasse 66, 38106 Braunschweig, Germany; 2 Laboratory for Emerging Nanometrology (LENA), TU Braunschweig, Langer Kamp 6, 38106 Braunschweig, Germany; 3 Department d’Electrònica, Universitat de Barcelona, c/Martí Franquès 1, 08028 Barcelona, Spain; 4 Department of Electronic and Computer Engineering, The Hong Kong University of Science and Technology, Clear Water Bay, Kowloon, Hong Kong, SAR China; 5 Fraunhofer Institute for Surface Engineering and Thin Films, Bienroder Weg 54E, 38108 Braunschweig, Germany; 6 School of Chemistry and Chemical Engineering, Jiangsu University, Xuefu Road 301, 212013 Zhenjiang, China

**Keywords:** H-TiO_2_ core-shell nanorods, Hydrogenation, Rapid thermal annealing, TEM/EELS, Optical absorption, PEC property

## Abstract

Recently, colored H-doped TiO_2_ (H-TiO_2_) has demonstrated enhanced photoelectrochemical (PEC) performance due to its unique crystalline core—disordered shell nanostructures and consequent enhanced conduction behaviors between the core-shell homo-interfaces. Although various hydrogenation approaches to obtain H-TiO_2_ have been developed, such as high temperature hydrogen furnace tube annealing, high pressure hydrogen annealing, hydrogen-plasma assisted reaction, aluminum reduction and electrochemical reduction etc., there is still a lack of a hydrogenation approach in a controlled manner where all processing parameters (temperature, time and hydrogen flux) were precisely controlled in order to improve the PEC performance of H-TiO_2_ and understand the physical insight of enhanced PEC performance. Here, we report for the first time a controlled and local rapid thermal annealing (RTA) approach to prepare hydrogenated core-shell H-TiO_2_ nanorods grown on F:SnO_2_ (FTO) substrate in order to address the degradation issue of FTO in the typical TiO_2_ nanorods/FTO system observed in the conventional non-RTA treated approaches. Without the FTO degradation in the RTA approach, we systematically studied the intrinsic relationship between the annealing temperature, structural, optical, and photoelectrochemical properties in order to understand the role of the disordered shell on the improved photoelectrochemical behavior of H-TiO_2_ nanorods. Our investigation shows that the improvement of PEC performance could be attributed to (i) band gap narrowing from 3.0 to 2.9 eV; (ii) improved optical absorption in the visible range induced by the three-dimensional (3D) morphology and rough surface of the disordered shell; (iii) increased proper donor density; (iv) enhanced electron–hole separation and injection efficiency due to the formation of disordered shell after hydrogenation. The RTA approach developed here can be used as a suitable hydrogenation process for TiO_2_ nanorods/FTO system for important applications such as photocatalysis, hydrogen generation from water splitting and solar energy conversion.

**Electronic supplementary material:**

The online version of this article (doi:10.1186/s11671-017-2105-x) contains supplementary material, which is available to authorized users.

## Background

Recently, H-doped TiO_2_ (H-TiO_2_) has triggered broad research interest due to its enhanced photocatalytic properties. Chen et al. reported that H-TiO_2_ nanoparticles were obtained by high-pressure hydrogen gas annealing. The nanoparticles contained an unique crystalline core and a disordered shell homo-interface which led to a narrowed band gap and enhanced photocatalytic behavior [1]. Wang et al. reported a new approach assisted by hydrogen plasma to synthesize H-doped black titania with core-shell nanostructures, superior to the high pressure hydrogenation process [2]. So far, the photocatalytic reports of H-TiO_2_ in literature are mainly limited to nanoparticle systems [3]. There are only a few investigations about H-TiO_2_ films on conductive substrates which can be used as photoanodes for *three-electrode photoelectrochemical* (PEC) studies [3]. Notably, highly oriented H-TiO_2_ nanorods (NRs) and nanotubes (NTs) have been demonstrated to be highly efficient photoanodes for solar light driven water splitting [4, 5]. Such an unidirectional nanostructure decouples the processes of light absorption and charge collection, which can benefit the charge carrier separation and transport [6–8]. However, the progress of hydrogen processing methods and their influence on the structural, optical, and photoelectrochemical behaviors of H-TiO_2_ is rarely reported due to lack of a practical hydrogenation method with excellent controllability on the processing parameters. Wang et al. reported a pioneer work of H-TiO_2_ nanorods grown on the fluorine-doped tin oxide (F:SnO_2_; FTO) substrate by high temperature hydrogen gas annealing in a tube furnace [4]. They studied the relation between the annealing temperature and photoelectrochemical properties. Due to the degradation issue of FTO substrate, the photocurrent density decreases at hydrogenation temperatures beyond 350 °C, an intrinsic relation between the annealing temperature and photoelectrochemical properties of H-TiO_2_ could not be indicated. The degradation issue of H-TiO_2_ nanorods/FTO material system will restrict its applications such as photocatalysis, hydrogen generation from water splitting and solar energy conversion.

**Fig. 1:**
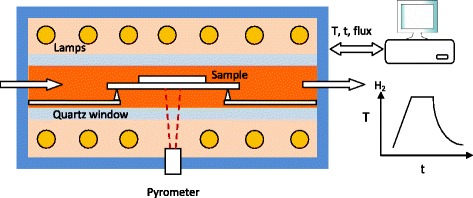
Schematic of hydrogenation of TiO_2_ nanorods by rapid thermal annealing (RTA) with controlled temperature recipe

Since the hydrogen treatment can strongly influence the structural and photocatalytic properties of H-TiO_2_ [9], a precise control of processing parameters (temperature, time, flux etc.) will play an important role to reproduce the core-shell structure and enhanced photocatalytic properties of H-TiO_2_ in order to identify the process–structure–PEC property relationship. It is known that rapid thermal annealing (RTA) is a standard semiconductor processing technique where the processing parameters can be precisely controlled by a PC [10, 11]. It has become essential to the fabrication of advanced semiconductors such as oxidation, annealing and deposition. It can provide fast heating and cooling to process temperatures of 300–1200 °C with ramp rates typically 10–250 °C/s, combined with excellent gas ambient control, allowing the creation of sophisticated multistage processes within one processing recipe. To our best knowledge, no work of H-TiO_2_ nanorods hydrogenated by RTA is reported till now. In comparison to the conventional hydrogen gas annealing, RTA allows the local thermal annealing on the samples. The RTA chamber is cooled down with cycled water, only the sample and sample holder (usually Si wafer) are locally heated by a set of infrared lamps (Fig. 1). Furthermore, several sharp quartz tips are used to support the sample and Si sample holder in order to prevent the thermal loss. It is evident that RTA consumes less energy than that of conventional hydrogen gas annealing.

In this work, we report for the first time the use of the RTA method to successfully prepare H-TiO_2_ nanorods grown on FTO substrate. The relationship of the processing parameters and morphology with the optical and photoelectrochemical properties is further illustrated with systematic characterization.

## Methods

### Growth of Ordered TiO_2_ Nanorods on FTO (F:SnO_2_) Substrate

TiO_2_ nanorods were directly grown on the FTO substrate via a previously reported hydrothermal method [12–14]. Typically, 0.35 ml of tetra-tert-butoxy titanate (Ti(O^t^Bu)_4_) was dissolved in 30 ml of 6 M HCl, and the solution was transferred into a steel-lined Teflon autoclave, where a cleaned FTO substrate was placed. The autoclave was constant at 160 °C for 18 h and the coated FTO substrate was washed several times with deionized water and ethanol. To remove the chemical residues and improve the crystallinity of TiO_2_ nanorods and their electric contacts with FTO glass, the samples were annealed at 550 °C for 3 h in air.

### Hydrogenation of TiO_2_ Nanorods by Rapid Thermal Annealing

In comparison to conventional hydrogen treatment in a tube furnace, we applied rapid thermal annealing (RTA) to make use of the controlled temperature recipes. Figure 1 shows the standard configuration of a typical rapid thermal annealing system. The samples were placed on a silicon wafer and heated by infrared lamps in hydrogen atmosphere. The temperature was measured by a pyrometer and controlled by a PID loop. All processing parameters (temperature, time, and hydrogen flux) were controlled precisely by a programmable recipe; therefore, a reproducible hydrogenation on TiO_2_ nanorods could be realized. For our experiments we chose a hydrogen flux of 50 sccm, 1 bar pressure, a temperature ramping rate of 5 °C/s, and the annealing temperatures of 350, 400, and 450 °C for 1 h each.

### Characterizations of the Samples

The H-TiO_2_ nanorods were investigated by using a scanning electron microscope, a X-Ray diffractometer, an optical absorption spectrometer and a high resolution transmission electron microscope (HR-TEM) Jeol J2010F FEG operating at 200 kV and coupled to a Gatan GIF filter for EELS analysis.

### Photoelectrochemical (PEC) Studies

A Newport solar simulator 150 W Xe lamp with AM 1.5G filter acted as a light source for the PEC test. The lamp power was adjusted using a reference silicon solar cell to obtain 100 mW/cm^2^ (1 sun). All J-V curves were recorded with a Princeton Applied Research 2273 potentiostat using 1 M KOH as electrolyte in three-electrode configuration (Ag/AgCl in 3 M KCl as a reference electrode and platinum wire as a counter electrode). The IPCE were performed based on Oriel QE-PV-SI (Newport Corporation) and electrochemical workstation (CHI660E, China). The M-S measurements were performed on the electrochemical workstation (Gamry, USA).

## Results and Discussion

**Fig. 2:**
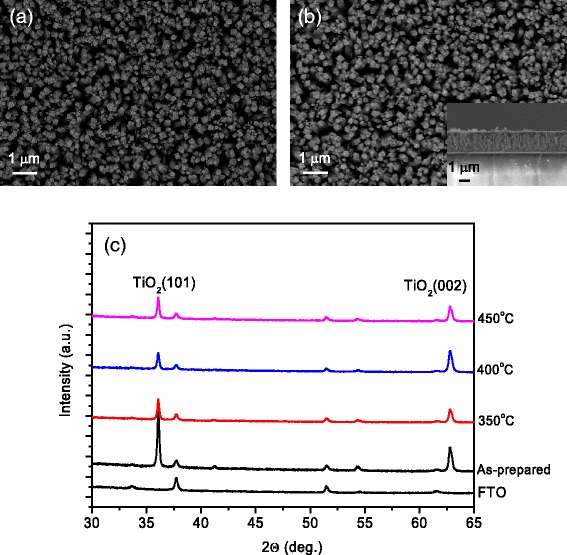
SEM images of (**a**) as-prepared TiO_2_ and (**b**) H-TiO_2_ nanorods treated at 400 °C. **c** XRD patterns of H-TiO_2_ nanorods treated at different temperatures

To study the possible change of morphology and crystal phase of H-TiO_2_ nanorods, scanning electron microscopy (SEM) images and X-ray diffraction (XRD) spectra were collected. Figure 2b shows the in-plane and cross-sectional SEM images of H-TiO_2_ nanorods treated at 400 °C. No changes of morphology and alignment are observed before and after RTA treatment. The nanorods show a good vertical alignment and similar length indicating high quality of H-TiO_2_ nanorod arrays. Figure 2c shows the XRD patterns of H-TiO_2_ nanorods treated at 350, 400, and 450 °C. The (101) and (002) diffraction peaks confirmed the good alignment of TiO_2_ nanorods on the FTO substrate [12–14]. After the hydrogenation, the intensities of (101) and (002) diffraction decrease indicating the generation of disordered shell around the TiO_2_ nanorods. In comparison to the (101) peak, the intensity of (002) decreases more when the annealing temperature increases. It indicates that the tips of TiO_2_ nanorods are treated stronger than the sidewalls near to the FTO substrate.

**Fig. 3:**
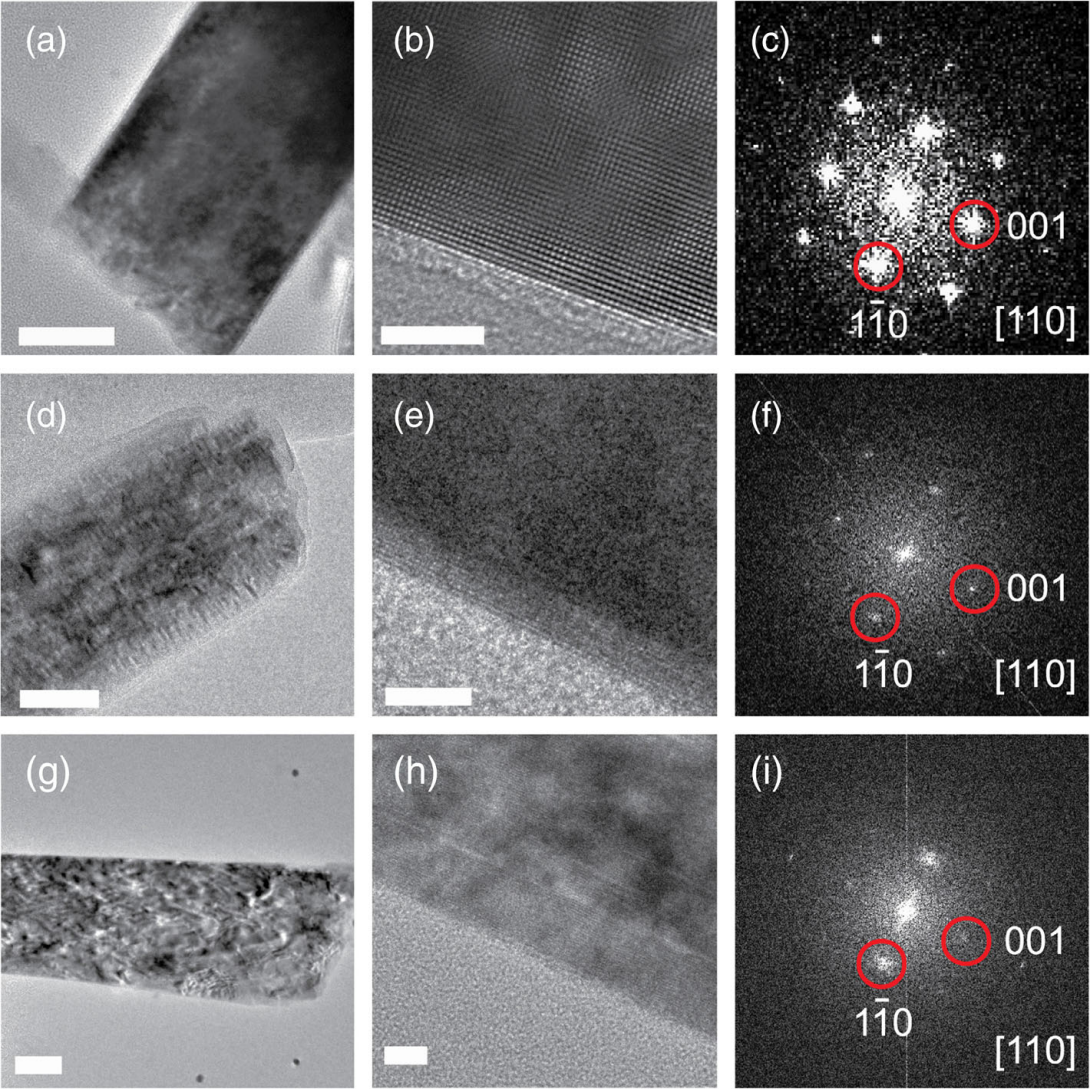
TEM, HR-TEM and FFT images of (**a**–**c**) as-prepared TiO_2_ nanorods and H-TiO_2_ treated at 400 °C (**d**–**f**) and 450 °C (**g**–**i**). The *scale bar* of Figs. 3 **a**, **d**, **g** is 50 nm whereas the scale bar of Figs. 3 **b**, **e**, **h** is 5 nm

The microstructures of single H-TiO_2_ nanorod and the outmost disordered shell were further investigated by high-resolution transmission electron microscopy (TEM). Figure 3 shows the TEM images of as-prepared and H-treated TiO_2_ nanorods. The as-prepared TiO_2_ nanorods are confirmed to be single crystalline (Figs. 3a–c) whereas the H-treated TiO_2_ nanorods show a single crystalline core and disordered shell heterostructure [15]. The amorphous shell of the sample treated at 400 °C (Figs. 3d–f) is ca. 4 nm whereas the shell of the sample-treated at 450 °C (Figs. 3g–i) becomes thicker and defective.

The optical absorption spectra of as-prepared TiO_2_ and H-TiO_2_ were recorded in order to study the modified optical absorption behavior of H-TiO_2_. Additional file 1: Figure S1a shows the small red-shift of band edge and gradual increase of visible absorption with increasing the annealing temperature. The Tauc plot (Additional file 1: Figure S1b) shows the band gap narrowing from 3.0 to 2.9 eV. The disordered shell contains a lot of oxygen vacancies and hydrogen atoms fill up partially in positions of oxygen vacancies which is assumed to result in the slight deformation of the rutile lattice cells and the band structure [16]. It is noted that the as-prepared TiO_2_ nanorods show the increased visible absorption in the range of 400–850 nm (Additional file 1: Figure S1a). This is due to the multi-step scattering of photons between the 3D nanorods. The visible absorption in the range of 400–850 nm of H-TiO_2_ nanorods increases further with increasing hydrogenation temperature. It could be attributed to the rough and porous shells and the altered absorption properties of the disordered shell of H-TiO_2_ nanorods to trap more visible photons.

**Fig. 4:**
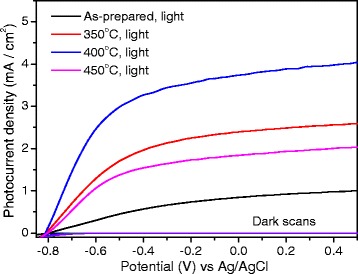
J-V *curves* of pristine TiO_2_ and H-TiO_2_ nanorods in 1 M KOH solution in the dark and under solar illumination (light)

To evaluate the photoelectrochemical behavior of H-TiO_2_ nanorods, J-V and IPCE measurements were performed in a three-electrode electrochemical system. Figure 4 shows that H-TiO_2_ nanorods exhibit a significant change in PEC performance with a maximum photocurrent density after hydrogen treatment at 400 °C. The photocurrent density of H-TiO_2_ treated at 400 °C saturates at a lower potential of −0.4 V vs Ag/AgCl whereas the density of as-prepared TiO_2_ saturates at −0.2 V vs Ag/AgCl. The photocurrent density of H-TiO_2_ treated at 400 °C reaches ca. 3.7 mA/cm^2^ at 0.23 V vs Ag/AgCl, which is more than four times larger than that of the as-prepared TiO_2_. The corresponding photoconversion efficiency is shown in Additional file 1: Figure S2.

**Fig. 5:**
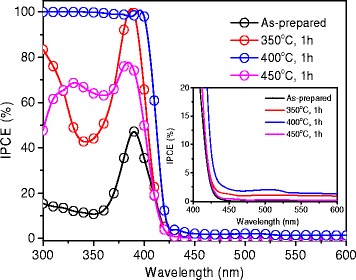
IPCE spectra of pristine TiO_2_ and H-TiO_2_ nanorods measured at the bias potential 0.23 V vs. Ag/AgCl

To clarify the influence of annealing temperature on the photoactivity, IPCE measurements were performed on the as-prepared and H-treated samples at 0.23 V vs Ag/AgCl (Fig. 5). IPCE can be expressed by the equation (eq. 1):

1 $$ IPCE=\frac{1240{J}_p}{\uplambda {J}_{\uplambda}}\mathrm{x}100\% $$

where *J*_p_ is the measured photocurrent density at a specific wavelength, *λ* is the wavelength of incident light, and *J*_*λ*_ is the measured irradiance at a specific wavelength. Figure 5 shows that the as-prepared TiO_2_ exhibits a maximum of about 45% IPCE at a wavelength of around 390 nm, consistent with literature data of rutile TiO_2_ nanorods. It is interesting to note that the IPCE of H-TiO_2_ shows an overall enhancement in the 300–600 nm range. The IPCE curves of H-TiO_2_ have three interesting features: (i) large increase of IPCE in the UV range from 300 to 400 nm; (ii) The band edge is red-shifted from 410 to 425 nm which is also observed in optical absorption spectra (Additional file 1: Figure S1); (iii) The IPCE of H-TiO_2_ samples show enhancement of IPCE in the visible (400–600 nm) range, in comparison to that of as-prepared samples. The 400 °C treated sample shows the best IPCE values in the range of 300–600 nm.

**Fig. 6:**
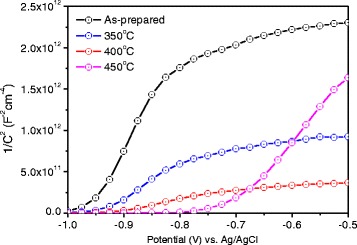
Mott-Schottky plots of pristine TiO_2_ and H-TiO_2_ nanorods

The influence of annealing temperature on H-TiO_2_ was further studied by Mott-Schottky (M-S) plots (Fig. 6). It is expected that the slope of H-TiO_2_ decreases with increasing annealing temperature indicating the increased donor density and conductivity according to the Mott − Schottky equation (eq. 2)

2 $$ {N}_d=\left(\frac{2}{{\mathrm{e}}_0{\upvarepsilon}_0{\upvarepsilon}_{\mathrm{r}}}\right){\left[\frac{d\left(1/{C}^2\right)}{ d V}\right]}^{-1} $$

where N_d_ is the donor density, e_0_ is the electron charge, ε_r_ is the dielectric constant of TiO_2_ nanorods, ε_0_ is the permittivity of vacuum, and V is the applied bias voltage. The calculated donor densities of H-TiO_2_ are 5.2 ± 0.1 × 10^17^ cm^−3^ (350 °C), 2.1 ± 0.1 × 10^18^ cm^−3^ (400 °C) and 3.0 ± 0.2 × 10^17^ cm^−3^ (450 °C) which are higher than that of the as-prepared TiO_2_ (1.5 ± 0.1 × 10^17^ cm^−3^) [17]. The 450 °C treated sample shows abnormal M-S plot which may be attributed to the more defective structures which is observed in the TEM image (Fig. 3g). Additionally, the flat band potentials (*V*_fb_) of as-prepared and H-TiO_2_ were determined by the extrapolation of straight line to the abscissa in the Mott-Schottky plots (Fig. 6). The V_fb_ of H-TiO_2_ treated at 350 °C is similar to that of as-prepared TiO_2_, however, the V_fb_ was observed to decrease from −0.95 V (pristine TiO_2_) to −0.97 V for the H-TiO_2_ treated at 400 °C. The negative shift of V_fb_ after RTA hydrogenation at 400 °C could be attributed to the substantially increased donor density (Additional file 1: Table S1), which could consequently shift the Fermi level (E_F_) of H-TiO_2_ towards to the conduction band (E_c_). The V_fb_ was observed to increase from −0.95 V (pristine TiO_2_) to −0.71 V for the H-TiO_2_ treated at 450 °C. It could be due to the inhomogeneous defective structure (Fig. 3g) and decreased donor density (Additional file 1: Table S1).

**Fig. 7:**
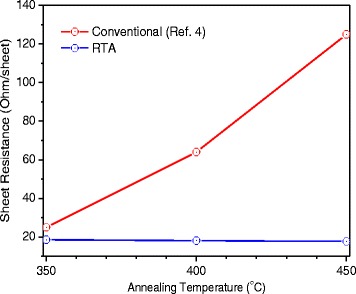
Sheet resistances of FTO and RTA treated FTO substrates compared with literature

To study the possible degradation of the FTO electrode in the RTA process, the sheet resistance of FTO treated at 350, 400, and 450 °C was measured by the four-probe method. In comparison to untreated FTO (~18.13 Ohm/sheet), the sheet resistances of the FTO after RTA treatment are unchanged (Fig. 7). Wang et al. reported the investigation of H-TiO_2_ nanorods by hydrogen gas annealing in a furnace tube and observed the degradation of the FTO layer when the temperature was higher than 350 °C (Fig. 7) [4]. In that study, it was difficult to study the effect of temperature-dependent hydrogen gas treatment due to the degradation issue of FTO layer. As no FTO degradation is observed in RTA process, the relation between RTA processing temperature, H-TiO_2_ structure and its properties can be discussed here in order to understand the mechanism of enhanced PEC performance. Our results could be related to the following factors:

(i) 3D morphology of H-TiO_2_ nanorods

**Scheme 1:**
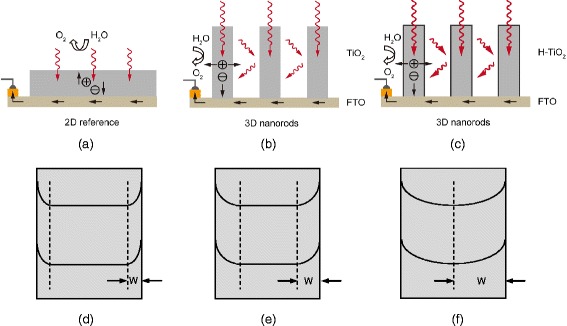
Light absorption and carrier charge transport in (**a**) 2D TiO_2_ film; (**b**) untreated 3D TiO_2_ nanorods and (**c**) 3D H-TiO_2_ nanorods, the outmost disordered shell is drawn with *black line* and the enhanced light scattering is marked with thick *arrows*. Relation between the diameter d of nanorods and the depletion region width W: (**d**) d > > W; (**e**) d > W and (**f**) d ~ W

In comparison to 2D films, 3D nanorods with subwavelength-scale induce the light-trapping effect [18–21]. When the light is incident on the surface of 3D nanorods, the photons will be reflected back and forth between the nanoscale gaps resulting in an increase of light absorption (Scheme 1). Higher optical absorption and lower reflection are the important factors in achieving higher photocurrent density. Cho et al. compared the current density with the best data of 2D and 3D TiO_2_ from literature [22]. It is evident that 3D TiO_2_ can achieve the photocurrent density 1 mA/cm^2^, three times higher than that of 2D TiO_2_ (ca. 0.3 mA/cm^2^) because 3D TiO_2_ can simultaneously offer larger surface area with electrolyte, higher optical absorption and shorter diffusion path length of holes from bulk via the surface into the electrolyte than those of 2D TiO_2_.

Furthermore, the optical spectra of H-TiO_2_ (Additional file 1: Figure S1) show a band gap narrowing and an enhanced visible absorption after the RTA hydrogenation. Cho et al. prepared branched TiO_2_ nanorods and co-doped C, W-TiO_2_ to achieve an enhancement of the visible optical absorption and PEC performance [22, 23]. It is evident that the 3D surface of TiO_2_ nanorods will be used not only for morphology change, but also for the change of optical absorption via chemical doping or hydrogenation in our work.

(ii) The role of disordered shell (TiO_2-x_H_y_)

The HR-TEM results show that the thickness of disordered shell increases with increased annealing temperature which is rarely reported in literature (Fig. 3). The EELS line-scan (Additional file 1: Figure S3) shows the O/Ti ratios across a single nanorod of as-prepared TiO_2_ and H-TiO_2_ treated at 400 °C, respectively. The O/Ti ratio of as-prepared TiO_2_ remains constant whereas the O/Ti ratio of H-TiO_2_ treated at 400 °C decreases gradually in the ca. 4 nm range of disordered shell. Recently Lü et al. prepared TiO_2_ homo-junction films consisting of an oxygen-deficient amorphous layer on top of a highly crystalline layer in order to simulate the crystalline core-disordered shell configuration of black TiO_2_ nanoparticles [24]. They observed that metallic conduction was achieved at the crystalline − amorphous homo-interface via electronic interface reconstruction, which may be the main reason for the enhanced electron transport of black H-TiO_2_. Since the composition of the disordered shell is important to understand the crystalline core-disordered shell homo-interface, detailed structural analysis will be performed in future studies.

We compared the IPCE data with literature in order to understand the improved PEC performance. Wang et al. reported nearly 100% IPCE above band-gap and less than 0.5% IPCE in the visible light range for the best samples treated at 350 °C [4]. Yang et al. reported ca. 80% IPCE above band-gap and less than 1.5% IPCE in the visible range [25]. Our IPCE spectrum of H-TiO_2_ treated at 400 °C shows nearly 100% IPCE in the UV range and ca. 2.5% IPCE in the visible range, additionally the band edge is red-shifted indicating a bandgap narrowing from 3.0 to 2.9 eV. This results in higher photocurrent density during operation, in comparison to literature.

The influence of the disordered shell on the PEC performance could be: *(i) efficient electron – hole separation:* Fabrega et al. studied the relationship between the donor density (N_d_) and the corresponding depletion region width (W) [17]. If the donor density is too high, the depletion region width is more localized nearby the nanorods surface which results in lower electron–hole separation efficiency (Scheme 1d). Therefore the control and optimization of donor density by hydrogenation will play a key role to achieve high PEC performance. Additional file 1: Table S1 shows the N_d_ values as a function of RTA annealing temperatures. It is evident that the donor density initially increases with increasing annealing temperature up to 400 °C. The depletion region width of 400 °C treated sample is ca. 78 nm which is close to the diameter of TiO_2_ nanorods. It means that the H-TiO_2_ nanorods are fully depleted and the electron–hole separation takes place in the whole nanorods (Scheme 1f). For higher temperatures the increasing disorder leads to a decrease in the PEC properties indicating an optimum treatment temperature at 400 °C. *(ii) efficient hole injection:* Pesci et al. performed transient absorption (TA) to study the lifetime of photo-generated holes [26]. Effective suppression of microsecond to seconds charge carrier recombination could be the key factor to improve the photoelectrochemical activity of H-TiO_2_. In comparison to the diameter of TiO_2_ nanorods, the less than 5 nm thick disordered shell is more like a surface co-catalyst [27, 28] which can (i) remove the surface electron trapping states; and (ii) promote hole injection into the electrolyte.

(iii) Hydrogenation issues of H-TiO_2_ nanorods/FTO system

In recent years, one-dimensional (1D) TiO_2_ nanorod arrays on FTO substrate received broad interest due to their utilization in important applications such as photocatalysis, hydrogen generation from water splitting and solar energy conversion [3]. It is clear that TiO_2_ nanorods/FTO system is not suitable for high temperature hydrogenation. The decreased conductivity of the FTO substrate is due to the formation of Sn metal upon reductive high temperature hydrogenation, the photocurrent of H-TiO_2_ nanorod photoanode is limited by the decreased FTO conductivity [4]. Our investigation shows that RTA approach is better than the conventional hydrogen gas annealing and could be a suitable solution for the hydrogenation of TiO_2_ nanorods / FTO system. Here, the H-TiO_2_ nanorods/FTO system without *FTO degradation* by RTA treatment could provide better performance in the above-mentioned applications [3].

The physical insight for the decreased PEC performance of H-TiO_2_ upon high temperature hydrogenation is not entirely understood. Leshuk et al. proposed that high temperature hydrogenation could be counterproductive to improving the photocatalytic activity of TiO_2_ because of its propensity to form bulk vacancy defects [29]. In comparison to TEM image of 400 °C treated sample, the H-TiO_2_ treated at 450 °C shows inhomogeneous contrast and more defective structures (Fig. 3g) indicating *strong structure destruction of TiO*_*2*_ during the hydrogenation process at higher temperature. It is clear that a controlled and suitable hydrogenation process is required for the development of H-TiO_2_ systems because their PEC properties are strongly determined by the art of treatment.

## Conclusions

In summary, we presented a controlled and local RTA hydrogenation method to treat TiO_2_ rutile nanorods and studied the effects of annealing temperature on the structural, optical and PEC properties of H-TiO_2_ nanorods. Our investigation shows that the improvement of PEC performance could be attributed to (i) band gap narrowing from 3.0 to 2.9 eV; (ii) improved optical absorption in the visible range induced by the three-dimensional (3D) morphology and rough surface of the disordered shell; (iii) increased proper donor density; (iv) enhanced electron–hole separation and injection efficiency due to the formation of disordered shell after hydrogenation. As there is an optimum of the PEC properties with respect to the hydrogenation process, the RTA method with its precise control over the processing parameters could be used as one of the standard hydrogenation processes for large-scale industrial applications. The features of RTA and conventional hydrogen gas annealing are summarized in Table 1. It indicates that RTA is more efficient than the conventional hydrogen gas annealing.

**Table 1:** RTA vs. conventional hydrogen gas annealing

Hydrogenation methods	Conventional hydrogen gas annealing	RTA
Chamber	Small sized samples Hot wall High energy consumption	Small and large scaled samples (<4”) Cool wall Low energy consumption → Local heating and soft treatment
Ramping/cooling time	~ Several hours	~ Several seconds or minutes
Holding time	~0.5-1 h	~1 h
Sheet resistance of FTO	Increased with increased annealing temperatures	Unchanged with increased annealing temperatures
Achieved photocurrent density (mA/cm^2^) at 0.23 V versus Ag/AgCl	2.5 (Ref.4) 2.9 (Ref.15)	3.7 (This work)

## Additional file

Additional file 1: Table S1. Donor density (N_d_), flat band potential (V_fb_) and depletion region width (W) of pristine TiO_2_ and H-TiO_2_ nanorods calculated from the Mott-Schottky plots. **Figure S1.** (a) Optical absorption spectra of pristine TiO_2_ and H-TiO_2_ nanorods. (b) Tauc plots of optical absorption curves for pristine TiO_2_ and H-TiO_2_ nanorods. **Figure S2.** Photoconversion efficiency of pristine TiO_2_ and H-TiO_2_ nanorods. **Figure S3.** The O/Ti ratio distribution along the nanorod diameter (a) pristine TiO_2_ and (b) H-TiO_2_ nanorods treated at 400 °C. The O/Ti ratio is estimated using EELS spectra taken from a cross-line shown in the TEM image. (DOCX 930 kb)

## Abbreviations

E_c_: Conduction band
EELS: Electron energy loss spectroscopy
E_F_: Fermi level
FTO: F:SnO_2_
HR-TEM: High-resolution transmission electron microscopy
IPCE: Incident photon-to-current efficiency
J-V: Photocurrent density–potential
M-S: Mott-Schottky
N_d_: Donor density
NRs: Nanorods
NTs: Nanotubes
PEC: Photoelectrochemical
PID: Proportional–integral–derivative
RTA: Rapid thermal annealing
SEM: Scanning electron microscopy
V_fb_: Flat band potential
XRD: X-ray diffraction
