# Supplementary material for: Enhanced Photoelectrochemical Behavior of H-TiO2 Nanorods Hydrogenated by Controlled and Local Rapid Thermal Annealing
Source: Nanoscale Res Lett. 2017 May 5;12:336. doi: 10.1186/s11671-017-2105-x (PMC5419951; doi:10.1186/s11671-017-2105-x)
Supplement: Additional file 1: Table S1. — Donor density (Nd), flat band potential (Vfb) and depletion region width (W) of pristine TiO2 and H-TiO2 nanorods calculated from the Mott-Schottky plots. Figure S1. (a) Optical absorption spectra of pristine TiO2 and H-TiO2 nanorods. (b) Tauc plots of optical absorption curves for pristine TiO2 and H-TiO2 nanorods. Figure S2. Photoconversion efficiency of pristine TiO2 and H-TiO2 nanorods. Figure S3. The O/Ti ratio distribution along the nanorod diameter (a) pristine TiO2 and (b) H-TiO2 nanorods treated at 400 °C. The O/Ti ratio is estimated using EELS spectra taken from a cross-line shown in the TEM image. (DOCX 930 kb) [file 11671_2017_2105_MOESM1_ESM.docx]

*Supporting Information*

Enhanced Photoelectrochemical Behavior of H-TiO_2_ Nanorods Hydrogenated by Controlled and Local Rapid Thermal Annealing

Xiaodan Wang,^†,‡,^* Sonia Estradé,^#^ Yuanjing Lin,^$^ Feng Yu, ^†,‡^ Lluis Lopez-Conesa,^#^ Hao Zhou, ^†,‡^ Sanjeev Kumar Gurram,^§^ Francesca Peiró,^#^ Zhiyong Fan,^$^ Hao Shen,^§,&,^* Lothar Schaefer,^§^ Guenter Braeuer,^§^ Andreas Waag^†,‡,^*

^†^Institute for Semiconductor Technology, TU Braunschweig, Hans-Sommer-Strasse 66, 38106 Braunschweig, Germany

‡Laboratory for Emerging Nanometrology (LENA), TU Braunschweig, Langer Kamp 6, 38106 Braunschweig, Germany

^#^Department d’Electrònica, Universitat de Barcelona, c/Martí Franquès 1, 08028 Barcelona, Spain

^$^Department of Electronic and Computer Engineering, The Hong Kong University of Science and Technology, Clear Water Bay, Kowloon, Hong Kong SAR, China.

^§^Fraunhofer Institute for Surface Engineering and Thin Films, Bienroder Weg 54E, 38108 Braunschweig, Germany

^&^School of Chemistry and Chemical Engineering, Jiangsu University, Xuefu Road 301, 212013 Zhenjiang, China

*Corresponding authors: w.xiaodan@tu-bs.de; hshen678@126.com; a.waag@tu-bs.de

**Table S1.** Donor density (N_d_), flat band potential (V_fb_) and depletion region width (W) of pristine TiO_2_ and H-TiO_2_ nanorods calculated from the Mott-Schottky plots.

**Figure S1.** (a) Optical absorption spectra of pristine TiO_2_ and H-TiO_2_ nanorods. (b) Tauc plots of optical absorption curves for pristine TiO_2_ and H-TiO_2_ nanorods.

**Figure S2.** Photoconversion efficiency of pristine TiO_2_ and H-TiO_2_ nanorods.

**Figure S3.** The O/Ti ratio distribution along the nanorod diameter (a) pristine TiO_2_ and (b) H-TiO_2_ nanorods treated at 400^o^C. The O/Ti ratio is estimated using EELS spectra taken from a cross-line shown in the TEM image.

**Table S1**

| Samples | N_d_ (cm^-3^) | V_fb_ (V vs. Ag/AgCl) | W (nm) at 0.23 V vs. Ag/AgCl |
| --- | --- | --- | --- |
| As-prepared | 1.50 x 10^17^ | -0.95 | 295 |
| 350 ^o^C | 5.19 x 10^17^ | -0.95 | 160 |
| 400 ^o^C | 2.15 x 10^18^ | -0.97 | 78 |
| 450 ^o^C | 2.98 x 10^17^ | -0.71 | 187 |

Since it is difficult to get the real active area of 3D H-TiO_2_ nanorods, we followed the suggestion from Fabrega’s work and assigned a donor density (1.50 x 10^17^ cm^-3^) to the as-prepared sample. The equation to calculate the depletion region width is given as-followed:

$$W=\sqrt{\frac{2\varepsilon_{0}\varepsilon_{r}\left| \phi_{\mathrm{SC}} \right|}{e_{0}N_{d}}}$$

Where N_d_ is the donor density, e_0_ is the electron charge, ε_r_ is the dielectric constant of TiO_2_ nanorods, ε_0_ is the permittivity of vacuum, ϕ_SC_ ≡ V − V_fb_ is the maximum potential drop in the depletion layer.

**Figure S1**

(a)

(b)

**Figure S2**

**
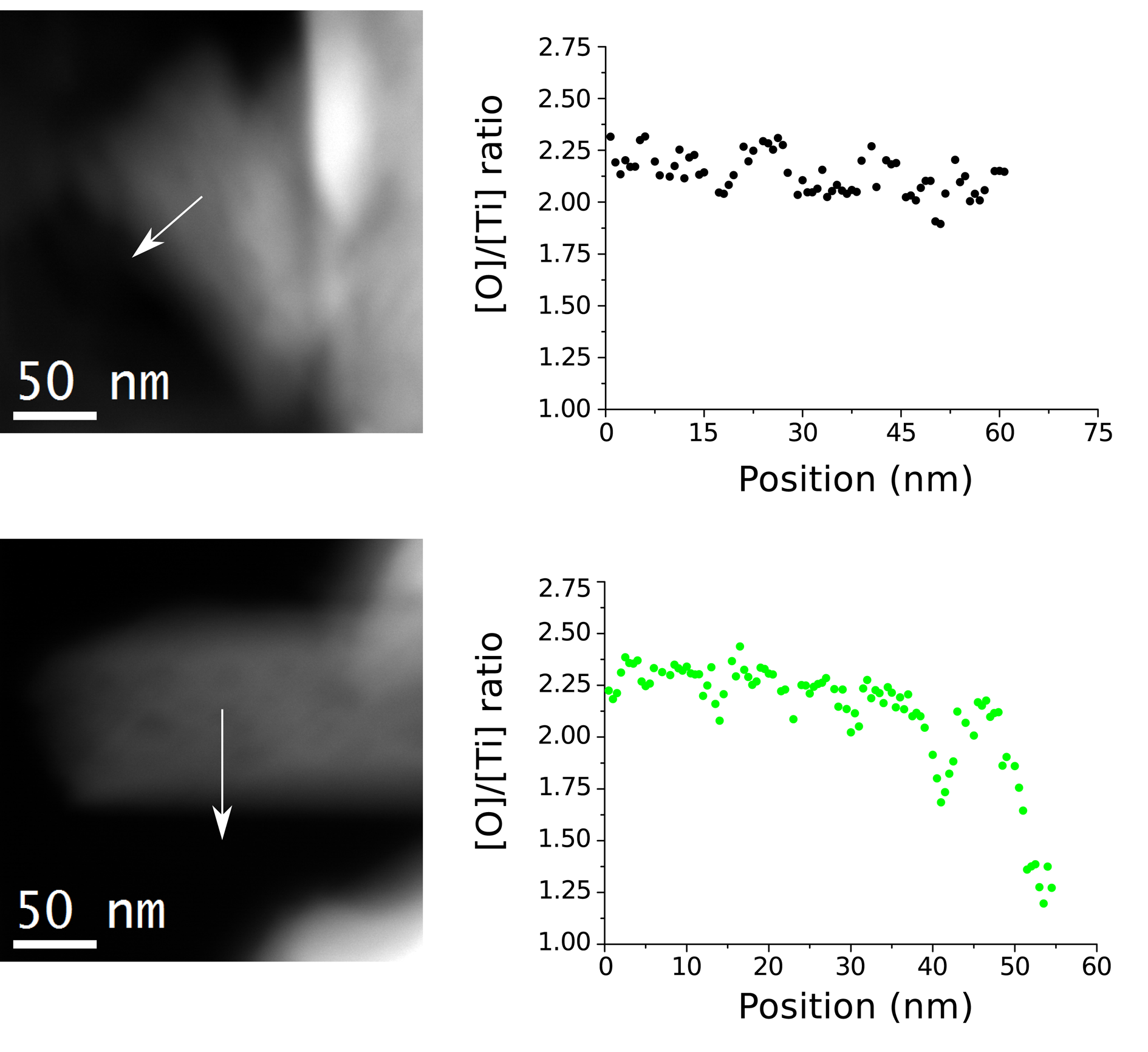
Figure S3**

(a)

(b)
